# Effects of Recruitment Maneuvers on Oxygenation and Intracranial Pressure in the Experimental ARDS Model

**DOI:** 10.5152/eurasianjmed.2022.21120

**Published:** 2022-10-01

**Authors:** Pelin Aydin, Mehmet Kizilkaya

**Affiliations:** 1 Department of Anesthesiology and Reanimation, Erzurum Regional Training and Research Hospital, Erzurum, Turkey; 2 Department of Anaesthesiology and Reanimation, Amasya University School of Medicine, Amasya, Turkey

**Keywords:** Acute respiratory distress syndrome, Recruitment maneuver, Oxygenation, Intracranial Pressure

## Abstract

**Objective:**

The most efficient recruitment maneuver (RM) for oxygenation in patients under mechanical circulatory support with acute respiratory distress syndrome (ARDS) has not been described yet. In this study, we have evaluated the effects of three recruitment maneuvers on oxygenation and intracranial pressure.

**Materials and Methods:**

20 sheep have been randomly grouped as follows: ARDS control, ARDS+TV(Tidal Volume), ARDS+CPAP(Continuous Positive Airway Pressure), ARDS+PEEP(Positive End Expiratory Pressure). Arterial blood gas tests have been done before ARDS, after ARDS, and 5,10, and 30 minutes after the maneuver. Intracranial pressures had been followed up.

**Results:**

There was a statistically significant increase in Pa02 (Partial arterial oxygen pressure) values in all groups 5 minutes after RM (*P* < 0,01). There was a statistically increase in Pa02 values in all groups 10 and 30 minutes after the RM (*P* < 0,01). TV group had significantly more increase in PaO2 increase at the 30th the other groups (*P* < 0,01). CPAP group had more increase in intracranial pressures just after the RM and at 5 and 10 minutes after the RM. This increase was statistically significant (*P* < 0,01). All groups had similar intracranial pressure values 30 minutes after the RM. There was no statiscally signifciant difference between the groups (*P* > 0,05).

**Conclusion:**

ICP monitorisation should be carried out in patients with ARDS while performing recruitment maneuvers.

**Main Points**

- This study focused to understand which one is more effective on oxygenation in lungs with acute respiratory distress syndrome (ARDS) by using 3 different non-traumatic recruitment maneuvers and to find the method that affects the intracranial pressure (ICP) least.
- Our results demonstrated that recruitment maneuver (RM) applications are quite useful in this particular, and they can significantly improve partial oxygen pressures.
- Recruitment maneuver can cause ICP increases even in cases where an ICP increase is not expected.
- Intracranial pressure should definitely be monitored while applying RM that can improve ARDS hypoxia.

## Introduction

Acute respiratory distress syndrome (ARDS) is the failure of the acute respiratory caused by noncardiogenic pulmonary edema. It starts as acute pulmonary edema and occurs with diffuse infiltration in both lungs.^1^ Acute respiratory distress syndrome develops for various reasons in roughly 200 000 people in the United States every year and 75 000 of those also die.^2^

There are many pulmonary and extrapulmonary causes leading to ARDS.^3^ Sepsis, among all ARDS causes, takes the first place. The foremost cause of pulmonary ARDS is the aspiration of stomach contents.^4^ Due to these reasons, intense inflammation occurs in ARDS which causes extensive alveolar injury. Permeability in pulmonary capillaries increases leading to the development of pulmonary edema. As a result lung compliance is reduced giving rise to serious clinical pictures that require mechanical ventilation support .^5^

Acute respiratory distress syndrome has no standard treatment method yet. Its treatment should be causal and needs supportive care.^6^ Lung-protecting mechanical ventilator (MV) support is the primary supportive care method. One of the treatment strategies in patients receiving MV support also includes the implementation of recruitment maneuvers (RM).^7^ The main purpose of RM; to open collapsed but openable lung areas in the damaged lung and to keep these areas open. Ultimately, it is to prevent hypoxemia resistant to oxygen therapy by reducing shunts.^8^ Today, many different RM have been described. It may be diversified in the way of breathing 1.5-2 times more of tidal volume (TV), increasing temporarily positive-end expiratory pressure (PEEP), increasing temporarily TV, applying high level continuous positive airway pressure (CPAP), high-frequency ventilation, the prone position (prone), and allowing the patient to spontaneous ventilation.^9,10^ There is no agreement on how often, to what pressure levels, how long, and in what way these maneuvers should be performed. It is uncertain which is the most effective method. Studies are not enough, either. Despite the advantages and disadvantages of each method, when evaluated together, it has been shown that all are effective in improving oxygenation and respiratory mechanics at certain rates and in reducing pulmonary atelectasis.^11^ Exclusively, unwanted effects may occur with these effects. Hemodynamic disturbances are the primary of these. It is also known to cause an increase in the risk of barotrauma/volutrauma, an increase in intracranial pressure (ICP), and a decrease in the clearance of alveolar fluid.^12^ Accordingly, new techniques that will not cause hemodynamic disturbances or complications that may occur due to pressure increase have been examined clinically and experimentally.^13^

We also in this study, unlike other studies, tried to understand which one is more effective on oxygenation in lungs with ARDS by using 3 different non-traumatic RMs and to find the method that affects the ICP least. Thus, when it is a matter of the development of ARDS in patients with traumatic and non-traumatic brain injury where ICP is often high, we aimed to reduce morbidity and mortality by applying RM which increases ICP the least.

## Materials and Methods

Approval with the decision number 2008/11 of Atatürk University Animal Experiments Local Ethics Committee was received for this study. Our study was done on 20 Karaman-type male sheep with an average weight of 35-40 kg in Atatürk University Experimental Research Center Laboratory (30/04/2008, decision number: 2008/11). Ethical regulations and all applicable rules in animal experiments were taken into consideration and NIH guidelines for use of laboratory animals were applied. Written informed consent was obtained from all participants who participated in this study.

The experimental subjects we used in our study were divided into 4 groups at random.

Groups:

ARDS control group: 5 sheep

ARDS + group increased TV: 5 sheep

ARDS + CPAP group: 5 sheep

ARDS + PEEP group: 5 sheep

Sheep fed a standard diet were hungered for 8 hours before the study. The skin areas where the electrodes were to be placed were shaved for electrocardiography (EKG) monitoring. It was monitored and heart rhythm was observed. Mask anesthesia was applied to the sheep with isoflurane and sedatized. A polyethylene 20 G IV cannula was placed in the vena femoralis in the inguinal regions and located with 3/0 atraumatic silk suture in order to be able to administer fluid and drug injections to sheep who were immobilized after sedation and had spontaneous ventilation. Ringer’s lactate solution was administered at a rate of 20 mL/kg/h as a maintenance fluid. Endotracheal intubation was performed after giving the 0.6 mg/kg rocuronium. An endotracheal tube of 9.0 mm was used for intubation. Volume-controlled mechanical ventilation was applied to the intubated sheep. The TV was set as 15 mL/kg, ventilation frequency 20/min, FiO_2_: 100%, and inspiratory–expiration rate of 1:2 in the mechanical ventilator. Peak inspiratory pressure was performed as 20 cmH_2_O.

In order to monitor arterial blood gas and take blood samples, femoral artery catheterization was performed. After the femoral artery was carefully isolated, the femoral artery was closed proximally and distally using 5/0 prolene suture. When the tidal volume is increased 3 times; Arterial blood gas analysis was performed before and after ARDS, 5, 10 and 30 minutes after the maneuver. pH, PaCO2, PaO2 values were measured, intracranial pressure was monitored. Inhalation anesthesia was administered with isoflurane gas from 1% to 2.5% for anesthesia maintenance.

Right jugular vein catheterization was performed. The sheep’s cervical areas were shaved. The puncture was performed with a set needle and full injector. After venous blood was aspirated, the injector was removed, preventing air leakage into it. The guide wire was inserted into the suture and carefully pushed forward without causing perforation or cardiac arrhythmias. After it was placed into the vein, the suture was removed completely, and the skin and subcutaneous tissues were dilated with a bougie. The catheter was placed into the vein over the guide wire. The guide wire was removed. Finally, the catheter was fixed with 3/0 atraumatic silk.

Intracranial pressure was monitored in all sheep. Intraparenchymal measurement technique was used. After the sheep’s heads were shaved, the midline was determined in their cranium. The 2 cm lateral of the midline at ear level was marked. The ICP catheter was placed after puncturing from the marked point with the burr hole. It was connected to the transducer and ICP monitoring was performed intraparenchymally.

Acute respiratory distress syndrome was created in all sheep used in the study. For this, 0.4 mL/kg 0.1N hydrochloric acid (HCl) was used. Half of the acid was slowly injected from the endotracheal tube intratracheally in the right lateral position and the other half was injected in the left lateral position.

First group ARDS + control group): All monitoring procedures were performed. Acute respiratory distress syndrome was created. Arterial blood gas analysis was performed before ARDS, immediately after ARDS, 5, 10, and 30 minutes after ARDS. pH, partial arterial carbon dioxide pressure (PaCO_2_), and partial arterial oxygen pressure (PaO_2__)_ values were measured, and ICP was monitored.

Second group ARDS + TV group: All monitoring procedures were performed. Acute respiratory distress syndrome was created. The method of increasing TV, which is one of the RMs, was used. In sheep with ARDS, TV was increased up to 2, 2.5, and 3 times at intervals of half-hour as planned beforehand. While the target TV was 3 times, pre- and post-ARDS, arterial blood gas analysis was performed 5, 10, and 30 minutes post-maneuver. pH, PaCO_2_, and PaO_2_ values were measured, and ICP was monitored.

Third group ARDS + CPAP group: All monitoring procedures were performed. Acute respiratory distress syndrome was created. The method of CPAP, which is one of the RMs, was used. Then, 10, 15, 20, 25, and 30 cmH_2_O CPAP was applied to the sheep with ARDS at intervals of half-hour as planned beforehand. The target 30 cmH_2_O CPAP was applied for 1 minute. Arterial blood gas analysis was performed before ARDS, after ARDS, 5, 10, and 30 minutes after the maneuver. pH, PaCO_2_, and PaO_2_ values were measured, and ICP was monitored.

Fourth group ARDS + PEEP group: All monitoring procedures were performed. ARDS was created. The method of PEEP, which is one of the RMs, was used. Then, 5, 10, 15, 20, 25, 30, 35, and 40 cmH_2_O PEEP was applied to the sheep with ARDS at intervals of half hour as planned beforehand. The target of 40 cm H_2_O PEEP was applied for 2 minutes. Arterial blood gas analysis was performed before ARDS, after ARDS, 5, 10, and 30 minutes after the maneuver. pH, PaCO_2_, and PaO_2_ values were measured, and ICP was monitored.

In statistical analysis, whether both groups had a difference between the previous and next values or not was used to analyze by matching test. Whether there was a difference between the means of the groups in each value or not was found using analysis of variance and Duncan tests. *P *< .05 was considered statistically significant and *P* < .01 was considered very significant. All data are given as mean standard deviation. Statistical analysis was made in Statistical Package for the Social Sciences software 13.0 package program.

## Results

**Figure 1.**
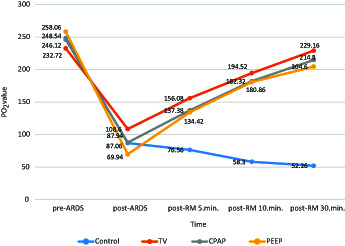
Change of the PaO_2_ values. PaO_2_, partial arterial oxygen pressure; ARDS, acute respiratory distress syndrome; TV, tidal volume; CPAP, continuous positive airway pressure; PEEP, positive end-expiratory pressure; RM, recruitment maneuver.

In our study, the ages, genders, and weights of sheep in all groups were the same. There was no statistically significant difference between PaO_2_ values in arterial blood gas analysis of all groups pre-ARDS (*P* > .05). In comparison with PaO_2_ values in arterial blood gas analysis in all of our acid-induced ARDS models and PaO_2_ values of pre-ARDS, we found a very significant decrease in the value of PaO_2_ (*P* < .01). PaO_2_ values of all groups increased statistically significantly (*P* < .01). Five minutes of post-RM was applied. No significant differences were observed between the groups. PaO_2_ values of all groups continued to increase in a statistically significant way at the 10th and 30th minutes of post-RM (*P* < .01). No difference was found between groups at 10 minutes of post-RM. However, PaO_2_ values were significantly increased in the TV group than in the PEEP group at the 30th minute (*P* < .01). There was a statistically very significant difference between PaO_2_ values of the control group and the other groups at the 5th, 10th, and 30th minutes of post-RM (*P* < .01). While a significant decrease was observed without an increase in the PaO_2_ value of the control group of post-ARDS, we obtained PaO_2_ values at an increasing level over time in all groups to that we applied RM (Table 1, Figure 1).

**Figure 2.**
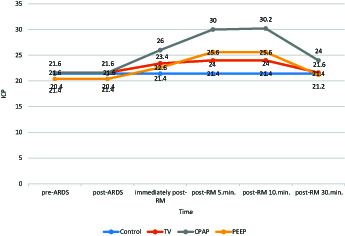
Change of the intracranial pressure values. ICP, intracranial pressure; ARDS, acute respiratory distress syndrome; TV, tidal volume; CPAP, continuous positive airway pressure; PEEP, positive end-expiratory pressure; RM, recruitment maneuver.

In our study, there was no statistically significant difference in ICP values between pre- and post-ARDS. The highest increase in the ICP values was measured. Immediately post-RM, 5th minute, and 10th minute post-RM were observed in the CPAP group, and this increase was evaluated as statistically very significant (*P* < .01). There was an increase in TV and PEEP groups, it was evaluated as statistically significant (*P *< .05). Intracranial pressure values in all groups at the end of 30 minutes of post-RM approached the baseline values; the difference was not found to be statistically significant (*P* > .05). In the control group, no significant difference was observed in ICP values of pre-ARDS, post-ARDS, immediately post-RM, and at the 5th, 10th, and 30th minutes of post-RM (Table 2, Figure 2).

**Figure 3.**
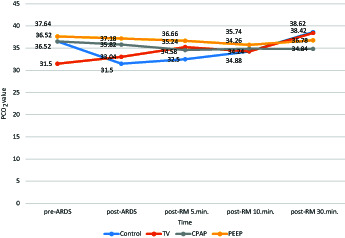
Change of the PaCO_2_ values. PaCO_2_, partial arterial carbon dioxide pressure; ARDS, acute respiratory distress syndrome; TV, tidal volume; CPAP, continuous positive airway pressure; PEEP, positive end-expiratory pressure; RM, recruitment maneuver.

In our study, the mean PaCO_2 _value of all groups of pre-ARDS decreased compared to the values of post-ARDS, but this was not found statistically significant (*P* > .05). PaCO_2_ values at 5, 10, and 30 minutes of post-RM approached the baseline values; this difference is not statistically significant (*P* > .05). In the control group no significant difference was observed in PaCO_2_ values of pre-ARDS, post-ARDS, and at the 5th, 10th, and 30th minutes of post-RM (Table 3, Figure 3).

**Figure 4.**
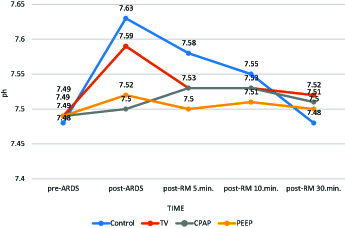
Change of pH values. pH, power of hydrogen; ARDS, acute respiratory distress syndrome; TV, tidal volume; CPAP, continuous positive airway pressure; PEEP, positive end-expiratory pressure; RM, recruitment maneuver.

When the pH values in arterial blood gases were analyzed, no statistically significant difference between the groups pre- and post-ARDS, at 5 minutes, 10 minutes, and 30 minutes of post-RM (*P* > .05) was found. In the control group, no significant difference was observed in pH values of pre-ARDS, post-ARDS, and at the 5th, 10th, and 30th minutes of post-RM (Table 4, Figure 4).

## Discussion

Acute respiratory distress syndrome is still a very serious problem accompanied by high morbidity and mortality despite the studies.^14^ Because of this reason, by protecting the lungs from damage, strategies targeting optimal oxygen and carbon dioxide levels have been developed.^15^ One of these strategies is the RM.^16^ Today, different RMs are applied at different durations and pressures within the scope of lung-reinforced mechanical ventilation strategies. Although many experimental and clinical studies have been carried out on this topic, it is still controversial as to which type of RMs should be applied, for how long, and at what pressure.^17^ When we analyzed these studies, we usually see that 1 or 2 types of maneuvers are compared to their effect only on oxygenation or only on ICP. We, in this study, more extensively compared the effects of 3 types of RMs on both oxygenation and ICP in an experimental model.

In animals with heavy ARDS, we saw that the impaired oxygenation improved dramatically after every 3 types of RM. While PaO_2_ values and hypoxia improved rapidly in the groups in which we applied RM, we found that PaO_2_ values decreased more over time and hypoxia deepened more in the control group. These findings show that we have created sufficient pressure at the alveolar level up to open the areas of collapse.

In normal respiratory physiology, there is a pressure difference of 5 cmH_2_O between transmural forces that widen the lungs and try to keep the airways closed, and this pressure difference keeps the airways open. This difference increases during inspiration and intrathoracic airways widen. The functional residual capacity must be greater than the closing capacity in order to avoid obstruction in the airways.^18^ If ARDS has developed in the lung, we cannot wait for the alveoli to open with normal pressures because the alveolar spaces are filled with a liquid rich in protein and debris. This liquid disrupts the structure and functions of the surfactant and as a result, alveoli collapses.^19^ Physiological shunt and deterioration in ventilation/perfusion ratio cause hypoxemia. Interstitial-alveolar edema and atelectasis cause a decrease in compliance.^20^ Under normal circumstances, although alveoli can be opened with TV, but when the above conditions occur, it is not possible to open all alveoli using only TV. The viscosity and high surface tension of the fluid in the collapsed lung prevents opening with TV. Whether collapsed alveole is damaged or not, it requires high airway pressure and time for opening.^21^ While lower pressures are sufficient to open collapsed airways, higher pressures should be applied to open collapsed alveoli.^22^ Various clinical and experimental studies are conducted to solve these problems. By the end of this study, it has been shown that hypoxia could be prevented by being 30-60 cmH_2_O of airway pressure in RMs that were found to be successful.^23-25^ Although the activity of the RM in ARDS models was very apparent and increases up to 50%, it has been shown that only 6% of the closed alveoli could be opened with the RM in patients with ARDS.^26^ In our study, we were able to provide sufficient oxygenation in all 3 of our RM groups. PaO_2_ values continued to gradually increase after applying RM. The values we measured at 30 minutes were almost the same as the pre-ARDS values in all 3 of the RM groups. The amount of pressure and the duration of pressure that we applied in all 3 RM groups show that we have created sufficient pressure at the alveolar level to open collapsed areas. In similar studies, it was reported that RM should be applied at sufficient pressure and for a sufficient time.^16^

In our study, we also measured the ICP values of the groups. In all 3 types of RM groups, we observed an increase in ICP values measured immediately post-RM and at the 5th and 10th minutes of post-RM. However, the increase in the group to which we applied CPAP was higher than the increase in the TV and PEEP groups. We consider that this increase in ICP in all 3 types of RM may be due to the decrease in cerebral venous return through the increase in intrathoracic pressure. Recruitment maneuver is a useful strategy that can improve oxygenation and optimize ventilation–perfusion nonadherence.^27^ However, it has been stated that RM can cause a significant increase in ICP in patients with altered cerebral autoregulation by disrupting jugular blood outflow, increasing intrathoracic pressure and central venous pressure, and preventing cerebral venous right return.^28^ In the studies, it has been stated that most of the lung-protective ventilation strategies may cause an increase in ICP.^29^ Because of these concerns, the clinical application of RM has been avoided until recently.^28,30^ Recently, there have been studies advocating that RMs can be administered in patients with brain damage with elevated ICP at baseline. ^31^ However, it cannot be said that it can be applied to these patients without worry. ICP and hemodynamic monitoring are required.^32^

In a study involving patients with subarachnoid hemorrhage accompanied by ARDS, the effect of CPAP and pressure control recruitment maneuver (PCRM) on ICP was analyzed. It has been stated that CPAP has increased ICP, while PCRM has not affected ICP.^33^ In our study, it was observed that there was an increase in ICP in all 3 types of RM, and this increase was the highest in the CPAP group. In an experimental study investigating the effect of PEEP application on ICP for different respiratory mechanics, in cases where ICP was normal in beginning, it has been shown that ICP increases significantly with increases in PEEP, and when PEEP increases in conditions where ICP is already high, ICP decreases significantly.^34^

As a result, for sure, our aim in ARDS treatment is to regulate ventilation, prevent hypercarbinia, and prevent hypoxia and tissue and organ damage that may develop secondary to hypoxia. When we examined our results, we found that RM is quite useful in this particular, and it can significantly improve partial oxygen pressures. In the ICP findings, which are another result of our study, we found that RM can cause ICP increase even in cases where ICP increase is not expected.

Because of this reason, we consider that ICP should definitely be monitored while applying RM which can improve ARDS hypoxia.

## Figures and Tables

**Table 1.** PaO_2 _Values of the Groups Pre- and Post-ARDS

**Mean ± Standard Deviation**	***P***	**Mean ± Standard Deviation**	***P***		**Mean ± Standard Deviation**	***P***	**Mean ± Standard Deviation**	***P***
	**Control**		** TV**		**CPAP**			**PEEP**	
	**Pre-ARDS**	248.54 ± 7.15	*.00	237.72 ± 15.09	*.00		246.12 ± 11.01	*.00	258.06 ± 38.18	*.00
**Post-ARDS**	87.06 ± 11.13	108.60 ± 12.98		87.94 ± 17.45			69.94 ± 21.54			
**Pre-ARDS**	248.54 ± 7.15	*.00	237.72 ± 15.09	*.00		246.12 ± 11.01	*.00	258.06 ± 38.18	*.01
**Post-RM 5 minutes**	76.56 ± 6.76		156.08 ± 6.63			137.38 ± 40.27		134.42 ± 34.93	
**Pre-ARDS**	248.54 ± 7.15	*.00	237.72 ± 15.09	*.04		246.12 ± 11.01	*.04	258.06 ± 38.18	*.01
**Post-RM 10 minutes**	58.30 ± 6.04		194.52 ± 21.46			182.32 ± 48.31		180.86 ± 26.26	
**Pre-ARDS**	248.54 ± 7.15	*.00	237.72 ± 15.09	*.04		246.12 ± 11.01	*.03	258.06 ± 38.18	*.01
**Post-RM 30 minutes**	52.26 ± 2.89		229.16 ± 10.89			214.80 ± 19.46		204.60 ± 23.88	

PaO_2_, partial arterial oxygen pressure; ARDS, acute respiratory distress syndrome; TV, tidal volume; CPAP, continuous positive airway pressure; PEEP, positive end-expiratory pressure; RM, recruitment maneuver.

*P < .05 intragroup comparison based on baseline value.

**Table 2.** ICP Values of the Groups Pre- and Post-ARDS

**Mean ± Standard Deviation**	***P***	**Mean ±** **Standard Deviation**	***P***	**Mean ± Standard Deviation**		***P***	**Mean ± Standard Deviation**	***P***
	Control		**TV**			**CPAP**		**PEEP**	
	**Pre-ARDS**	21.40 ± 2.07	*.00	21.60 ± 1.81	*.02	21.60 ± 2.50		*.00	20.40 ± 1.14	*.01
**İmmediately post-RM**	21.40 ± 2.07	23.40 ± 2.07		26.00 ± 1.58		22.60 ± 1.67				
**Pre-ARDS**	21.40 ± 2.07	*.00	21.60 ± 1.81	*.00	21.60 ± 2.50		*.00	20.40 ± 1.14	*.00
**Post-RM 5 minutes**	21.40 ± 2.07		24.00 ± 1.73		30.00 ± 3.53			25.60 ± 2.30	
**Pre-ARDS**	21.40 ± 2.07	*.00	21.60 ± 1.81	*.00	21.60 ± 2.50		*.00	20.40 ± 1.14	*.00
**Post-RM 10 minutes**	21.40 ± 2.07		24.00 ± 1.73		30.20 ± 3.70			25.60 ± 2.30	
**Pre-ARDS**	21.40 ± 2.07	*.00	21.60 ± 1.81	1.00	21.60 ± 2.50		.11	20.40 ± 1.14	.40
**Post-RM 30 minutes**	21.40 ± 2.07		21.60 ± 1.67		24.00 ± 3.39			21.20 ± 2.38	

ICP, intracranial pressure; ARDS, acute respiratory distress syndrome; TV, tidal volume; CPAP, continuous positive airway pressure; PEEP, positive end-expiratory pressure; RM, recruitment maneuver.

*P < .05 intragroup comparison based on the baseline values.

**Table 3.** Distribution of Mean PaCO_2 _Values of the Groups

	**Pre-ARDS PaCO_2_** 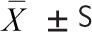	**Post-ARDS PaCO _2_** 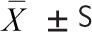	**Post-RM 5 minutes PaCO_2_** 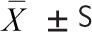	**Post-RM 10 minutes PaCO_2_** 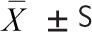	**Post-RM 30 minute PaCO _2_** 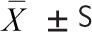
	**Control**	36.52 ± 4.15	31.50 ± 7.61	32.50 ± 4.64	34.24 ± 4.57	38.62 ± 1.59
**TV**	38.48 ± 6.52	33.04 ± 7.57	35.24 ± 6.93	34.26 ± 4.69	38.42 ± 4.29
**CPAP**	36.52 ± 7.17	35.82 ± 2.05	34.58 ± 2.85	34.88 ± 3.16	34.84 ± 2.27
**PEEP**	37.64 ± 9.41	37.18 ± 3.60	36.66 ± 7.76	35.74 ± 0.79	36.78 ± 1.01
***P***	.96	.41	.73	.90	.11

PaCO_2_, partial arterial carbon dioxide pressure; ARDS, acute respiratory distress syndrome; TV, tidal volume; CPAP, continuous positive airway pressure; PEEP, positive end-expiratory pressure; RM, recruitment maneuver.

**Table 4.** Distribution of Mean pH Values of the Groups

	**Pre-ARDS pH** 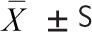	**Post-ARDS pH** 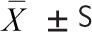	**Post-ARDS RM 5 minutes pH** 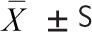	**Post-ARDS RM 10 minutes pH** 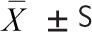	**Post-ARDS RM 30 minutes pH** 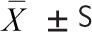
	**Control**	7.48 ± 0.09	7.63 ± 0.11	7.58 ± 0.07	7.55 ± 0.08	7.48 ± 0.01
**TV**	7.49 ± 0.11	7.59 ± 0.11	7.53 ± 0.11	7.53 ± 0.08	7.52 ± 0.06
**CPAP**	7.49 ± 0.10	7.50 ± 0.05	7.53 ± 0.07	7.53 ± 0.07	7.51 ± 0.05
**PEEP**	7.49 ± 0.10	7.52 ± 0.06	7.50 ± 0.12	7.51 ± 0.05	7.50 ± 0.02
***P***	.99	.12	.70	.87	.56

pH, power of hydrogen; ARDS, acute respiratory distress syndrome; TV, tidal volume; CPAP, continuous positive airway pressure; PEEP, positive end-expiratory pressure; RM, recruitment maneuver.
